# Structure and Dynamics of Ferroelectric Domains in Polycrystalline Pb(Fe_1/2_Nb_1/2_)O_3_

**DOI:** 10.3390/ma12081327

**Published:** 2019-04-23

**Authors:** Hana Ursic, Andreja Bencan, Uros Prah, Mirela Dragomir, Barbara Malic

**Affiliations:** 1 Electronic Ceramics Department, Jozef Stefan Institute, Jamova cesta 39, 1000 Ljubljana, Slovenia; andreja.bencan@ijs.si (A.B.); uros.prah@ijs.si (U.P.); mirela85.dragomir@gmail.com (M.D.); barbara.malic@ijs.si (B.M.); 2 Jozef Stefan International Postgraduate School, Jamova cesta 39, 1000 Ljubljana, Slovenia; 3 Department of Chemistry and Chemical Biology, McMaster University, Hamilton, ON L8S 4K1, Canada

**Keywords:** Pb(Fe_1/2_Nb_1/2_)O_3_, ferroelectric domain, piezoresponse force microscopy, monoclinic phase

## Abstract

A complex domain structure with variations in the morphology is observed at ambient temperature in monoclinic Pb(Fe_1/2_Nb_1/2_)O_3_. Using electron microscopy and piezoresponse force microscopy, it is possible to reveal micrometre-sized wedge, lamellar-like, and irregularly shaped domains. By increasing the temperature, the domain structure persists up to 80 °C, and then starts to disappear at around 100 °C due to the proximity of the ferroelectric–paraelectric phase transition, in agreement with macroscopic dielectric measurements. In order to understand to what degree domain switching can occur in the ceramic, the mobility of the domain walls was studied at ambient temperature. The in situ poling experiment performed using piezoresponse force microscopy resulted in an almost perfectly poled area, providing evidence that all types of domains can be easily switched. By poling half an area with 20 V and the other half with −20 V, two domains separated by a straight domain wall were created, indicating that Pb(Fe_1/2_Nb_1/2_)O_3_ is a promising material for domain-wall engineering.

## 1. Introduction

Pb(Fe_0.5_Nb_0.5_)O_3_ (PFN) is a multiferroic material that is paramagnetic at ambient temperature [1,2]. It belongs to the class of lead-based complex perovskites with the general formula Pb(B′*_x_*B″_1−*x*_)O_3_. While some compounds belonging to this class are relaxor ferroelectrics (or “relaxors” for short), such as Pb(Mg_1*/*3_Nb_2*/*3_)O_3_ and Pb(Zn_1*/*3_Nb_2*/*3_)O_3_ [3,4], PFN exhibits piezoelectric and ferroelectric properties at ambient temperature [5,6,7,8,9]. Namely, a piezoelectric coefficient d_33_ ≈ 140 pC·N^−1^, a coupling factor k_33_ ≈ 0.27, and a saturation polarisation ≈ 11 µC·cm^−2^ were reported [5]. However, the ferroelectric domains and their switching were not yet revealed. Until now, only one transmission electron microscopy (TEM) image showing wedge-shaped domains a few hundred nanometres in size [10] had been reported.

The domain structure was previously only investigated in PFN single crystals by optical [11] and TEM [12] microscopy. The micrometre-sized wedge domains [11,12] that coexist with tweed domains were observed at room temperature. The domain configuration was stable upon heating to ~50 °C. The wedge-shaped domains vanished at 80 °C, while the tweed domains became blurred at 90 °C and completely disappeared at 112 °C [12].

In polycrystalline samples, the ferroelectric domain structure was only studied in modified PFN. For Li-doped PFN, the piezoresponse force microscopy (PFM) study revealed stripe-like ferroelectric domains [13], but the PFM images were overlapped with the topographical cross-talk. Therefore, it is difficult to distinguish between the polishing lines and the domains. In 0.9PFN–0.1PbZrO_3_ ceramics, wedge-shaped domains were observed using electron microscopy [14]. 

In this study, we investigate the ferroelectric domains and their dynamics in polycrystalline PFN. The Rietveld refinement of the X-ray powder diffraction (XRD) shows that the samples are monoclinic. The domain structure is investigated using three complementary microscopies, i.e., PFM, TEM, and scanning electron microscopy (SEM). Each of these methods has their own limitations, but together they can describe the ferroelectric domain structure more comprehensively. The temperature evolution of the domain structure was performed with an in situ PFM experiment, revealing that the domain structure starts to disappear at around 100 °C, when crossing the Curie temperature, as determined from the dielectric permittivity vs. temperature curves. Furthermore, in this work, the emphasis is given also to the investigation of the domain-switching behaviour by applying a local DC electric field in PFM, resulting in an almost complete poling of the chosen area at ambient temperature. The local switching behaviour is supported by macroscopic measurements of polarisation versus electric field loop and the piezoelectric coefficient. 

## 2. Materials and Methods

For the synthesis of the PFN powder, PbO (99.9%, Sigma-Aldrich, St. Louis, MO, USA), Fe_2_O_3_ (99.9%, Alfa Aesar, Haverhill, MA, USA), and Nb_2_O_5_ (99.9%, Sigma-Aldrich) were used. The homogenised stoichiometric mixture (200 g) was mechano-chemically activated in a planetary ball mill (Retsch, Model PM 400, Haan, Germany) for 30 h at 300 min^−1^ using a tungsten carbide milling vial (volume 250 cm^3^) and milling balls (ball diameters 20 mm). The synthesised powder was milled in an attrition mill with yttria-stabilised zirconia (YSZ) milling balls (ball diameter 3 mm) in isopropanol for 4 h at 800 min^−1^. The powder was then uniaxially pressed into disks and further consolidated by isostatic pressing at 300 MPa. The powder compacts were reactively sintered in double alumina crucibles in the presence of a PFN packing powder with the same chemical composition to avoid possible PbO losses. The compacts were sintered at 1000 °C for 2 h in an oxygen atmosphere, as previously suggested in [6,15]. The heating and cooling rates were 2 °C·min^−1^. The density of the sintered pellets determined by Archimedes’ method was ~96% of the theoretical density [16]. 

For the microstructural analysis, the PFN samples were fractured for a fracture-surface examination, ground and polished using standard metallographic techniques for the polished-surface examination, and fine-polished (by a colloidal silica suspension with 0.04 µm-sized colloidal SiO_2_ particles for 1.5 h) followed by thermal etching at 750 °C for 5 min for the thermally etched surface examination. The microstructure was examined with a field-emission scanning electron microscope (FE-SEM, JSM-7600F JEOL Ltd., Tokyo, Japan) at 15 kV. For the stereological analysis, more than 340 grains were measured using the Image Tool Software [17], and the grain size was expressed as the Feret’s diameter [18]. A JEOL ARM 200 CF scanning–transmission electron microscope (STEM), equipped with Centurio energy-dispersive X-ray spectroscopy (EDXS) system, was used for the chemical and structural characterisation (Supplement S1). The TEM samples were prepared by mechanical grinding, dimpling and final Ar-ion milling [19].

The X-ray powder diffraction (XRD) pattern of the crushed PFN pellet was recorded using a PANalytical X’Pert PRO (PANalytical, Almelo, Netherlands) high-resolution diffractometer with Cu-K*α*_1_ radiation (*λ* = 1.54056 Å) and an X’Celerator detector. The XRD pattern was collected over the 2*θ* range of 15–120° with a step of 0.008° and an integration time of 100 s per step. In order to determine the phase structure, a Rietveld refinement analysis was performed (Supplement S2).

Prior to the PFM analysis, the samples were cut and polished using standard metallographic techniques. The samples were then heated to 600 °C for 1 h and then cooled back to room temperature with a cooling rate of 1 °C·min^−1^, to release the internal stresses formed in the material during cutting and polishing. The piezoresponse images were recorded with an atomic force microscope (AFM; Asylum Research, Molecular Force Probe 3D, Santa Barbara, CA, USA) equipped with a PFM module. A tetrahedral Si tip coated with Ti/Ir (Asyelec-01, AtomicForce F&E GmbH, Mannheim, Germany) with a curvature diameter of 30 ± 10 nm was applied to scan the sample surface. The spring constant and the resonance frequency of the cantilevers were 2 N·m^−1^ and 70 kHz, respectively. The electric field was applied through the sample. The out-of-plane amplitude and phase PFM images were measured in the dual AC resonance-tracking mode. The used frequencies for scanning the AC electric field were 310 and 350 kHz. The local hysteresis was measured in the switching-spectroscopy mode [20] with the following waveform parameters: The sequence of the increasing DC steps signal was 20 Hz with a maximum amplitude of 20 V, and the sequence of the triangle envelope was 0.1 Hz and an overlapping AC signal of 5 V and 350 kHz. Three cycles were measured in the off-loop mode. For the temperature evolution of the PFM response, a heater (Polymer heater, Asylum Research, Santa Barbara, CA, USA) was implemented. The in situ poling experiment was carried out using the PFM lithography mode and a square pattern (DC voltage ± 20 V).

For the electric measurements, the surfaces of ~200 μm-thick samples were covered by sputtered Cr/Au electrodes with diameters of 5 mm. The dielectric permittivity *ε*′ and the dielectric losses tan *δ* versus temperature were measured at 1, 10, and 100 kHz with a HP 4284 A Precision LCR impedance meter during cooling in the temperature range between 25 and 175 °C. The temperature-dependent polarisation (*P*) vs. electric field (*E*) measurements were made using the commercial setup Aix-PES (Aixacct Systems, Aachen, Germany). A bipolar sine wave with a frequency of 20 Hz was used as the input signal. The piezoelectric coefficient *d*_33_ was measured at 100 Hz of stress frequency using a standard Berlincourt piezometer (Take Control).

## 3. Results and Discussion 

**Figure 1:**
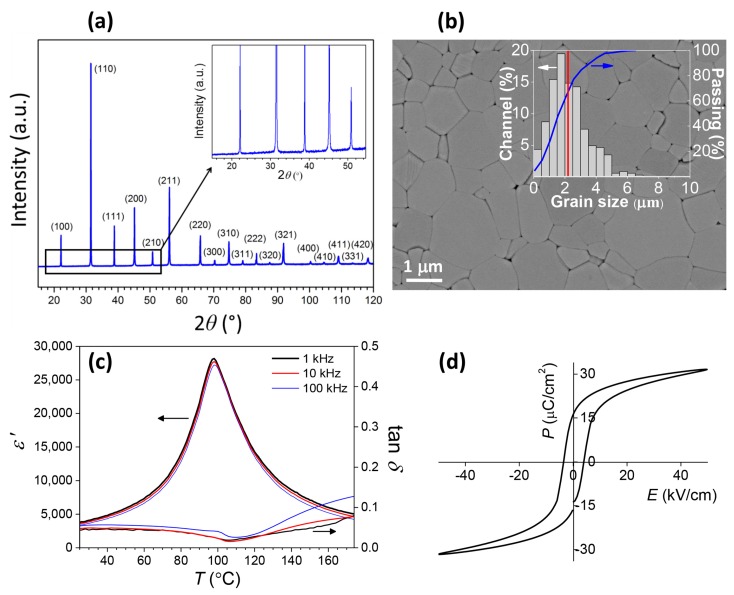
(**a**) Room-temperature X-ray powder diffraction (XRD) pattern of the Pb(Fe_0.5_Nb_0.5_)O_3_ (PFN) sample. The indexed peaks of the perovskite phase are shown in brackets (PDF card no. 032-0522 [16]). The inset shows an enlarged 2*θ* region from 15 to 55°. (**b**) Field-emission scanning electron microscope (FE-SEM) micrograph of the thermally etched surface. Inset: The grain size distribution together with the cumulative curve. (**c**) The dielectric permittivity *ε*′ and dielectric losses tan *δ* versus temperature. (**d**) Polarisation–electric field (*P*–*E*) hysteresis loop at 25 °C.

We begin by analysing the phase structure, microstructure, and dielectric, piezoelectric, and ferroelectric properties of the prepared PFN ceramics. In Figure 1a, the XRD pattern of a crushed ceramic pellet is shown. No secondary phases are observed, which is in agreement with the FE-SEM and TEM/EDXS analyses (Supplement S1). The FE-SEM micrograph of the thermally etched surface is shown in Figure 1b. Normal grain growth with a unimodal grain size distribution is observed, with an average grain size of 2.3 ± 1.2 µm. The ceramic consists of a monoclinic phase with the space group *Cm*, as determined by the Rietveld refinement (Supplement S2).

The temperature dependences of *ɛ*′ and tan *δ* are shown in Figure 1c. At ambient temperature, the *ɛ*′ is 3780 at 1 kHz. The peak permittivity *ɛ′_max_* of ~28,000 at 1 kHz appears at 98 °C (Curie temperature). At ambient temperature, the tan *δ* is 0.038 at 1 kHz. The *P*–*E* measurement at room temperature is shown in Figure 1d. The remnant polarisation (*P_r_*) and maximum polarisation (*P_max_*) are 16.5 and 31.1 µC·cm^−2^, respectively. The *ɛ*′, *P_r_*, and *P_max_* are slightly higher than the previously reported values [5,6,7,8,21,22,23]. The piezoelectric coefficient *d*_33_ is 140 pC·N^−1^, which is similar to the previously reported value [5].

**Figure 2:**
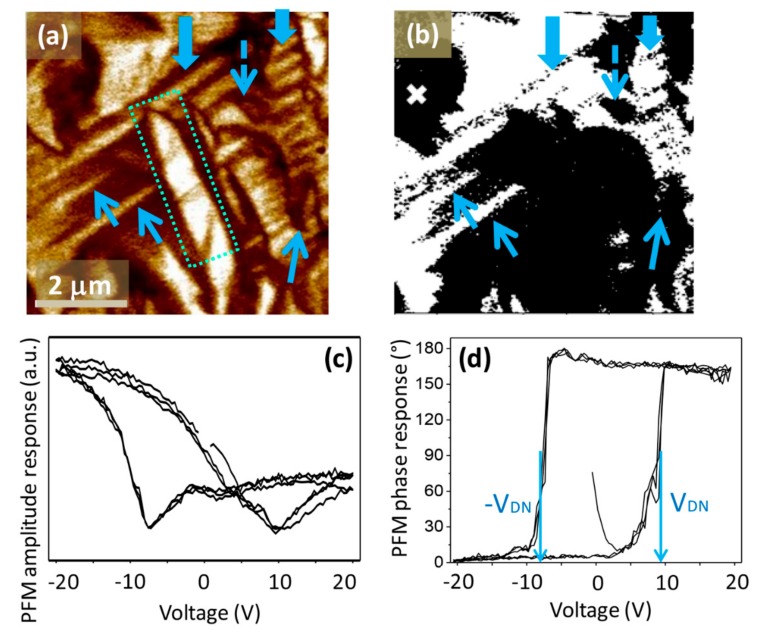
Piezoresponse force microscopy (PFM) out-of-plane (**a**) amplitude and (**b**) phase images (drive AC signal: 3 V, 350 kHz). The examples of wedge domains (thin solid arrows), lamellar-like domains (thick solid arrows), and irregularly shaped domains (dashed arrow) are shown. Local hysteresis loops; (**c**) amplitude, and (**d**) phase measured after scanning the PFM images in the spot marked by a cross in (**b**). The ±*V*_DN_ is marked by vertical arrows in the panel (**d**).

We continue by investigating the ferroelectric domain structure. At room temperature, a complex domain configuration with variations in the morphology and a characteristic length is observed, confirming the long-range ferroelectric order, which is in agreement with the piezoelectric and ferroelectric properties. The PFM out-of-plane amplitude and phase images are shown in Figure 2, where three different types of domains are observed. 

The first type is the wedge domains that were already observed, in both cases by TEM, in PFN polycrystalline samples [10] and single crystals [12]. In the former, the authors observed only wedge-like domains, while in the latter the wedge-shaped domains coexist with tweeds. The authors reported that the wedges are actually the boundaries between the tweed domains. 

**Figure 3:**
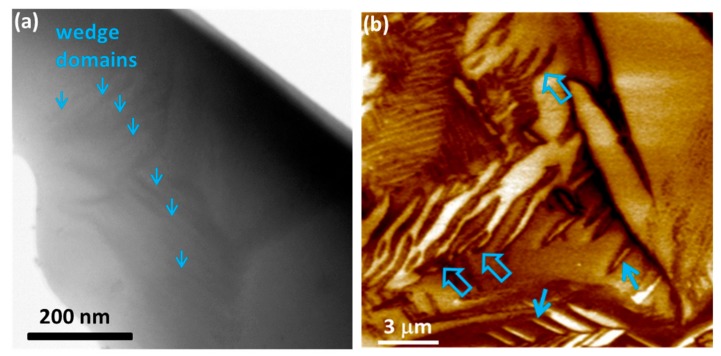
(**a**) Scanning–transmission electron microscope (STEM) image and (**b**) PFM out-of-plane amplitude image (drive AC signal: 6 V, 350 kHz) showing wedge domains. In (**b**), the domains with straight and curved domain walls (DWs) are marked with solid and thick open arrows, respectively.

In our case, the wedges appear overlapping, as can be seen in Figure 2 (marked by a dotted rectangle). Wedge domains, a few hundred nanometres in size, were observed also by STEM, as shown in Figure 3a. They can be further divided into two subgroups, either with flat or with curved domain walls (DWs), as demonstrated in Figure 3b. Both types of domains were already observed in the polycrystalline samples [10], while only curved DWs were detected in the single crystals [12]. Such wedge-type domains have previously been observed in some ferroelectric materials with a monoclinic structure [24,25].

The second type is the periodic or so-called lamellar-like domains (example shown in Figure 2), which would most probably develop in wedge domains, if not limited by the defects or grain boundaries. Such domains were previously observed in Li-doped PFN ceramics by PFM [13]. However, as mentioned in the introduction, it is difficult to distinguish between the polishing lines and the stripe-like domains. 

**Figure 4:**
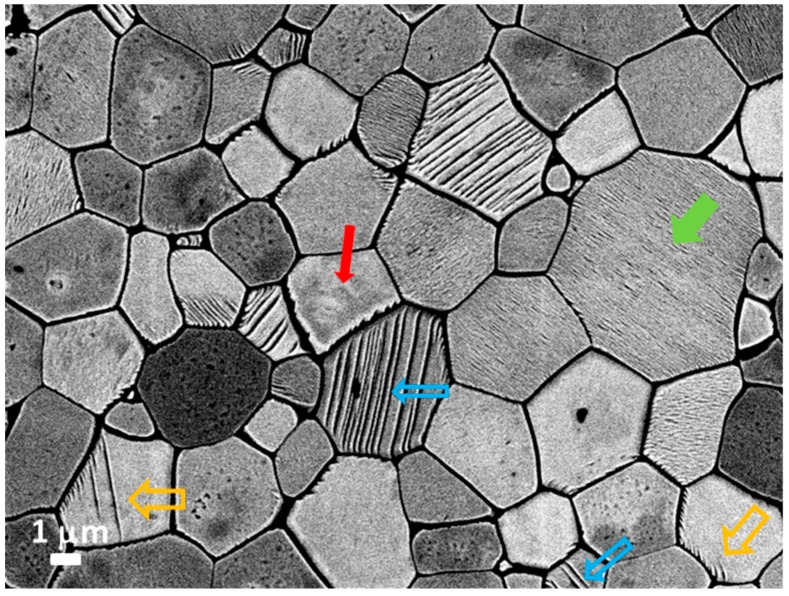
FE-SEM micrograph of polished and thermally etched surface of PFN sample showing micro-sized wedge (thick open orange arrows), irregularly shaped (thin solid red arrow), and lamellar-like (thin open blue arrows) domains. Domains, a few hundred nanometres in size (thick solid green arrow), are also observed.

The last type of ferroelectric domains observed in our samples are the irregularly shaped domains or so-called finger-print domains (example shown in Figure 2). This type has not been explicitly reported in previous studies of the PFN domain structure, but they can be observed in the PFM images of Li-doped PFN ceramics [13]. The irregular domains, sometimes called watermark domains, were also previously observed in other polycrystalline ferroelectrics, such as Pb(Sc_1/2_Nb_1/2_)O_3_ and BaTiO_3_ [26,27]. The presence of all three kinds of domains, i.e., wedge, lamellar-like, and irregularly shaped, was additionally confirmed by SEM imaging. In Figure 4, domains a few hundred nanometres in size are also observed, which is in agreement with the STEM investigation shown in Figure 3a.

**Figure 5:**
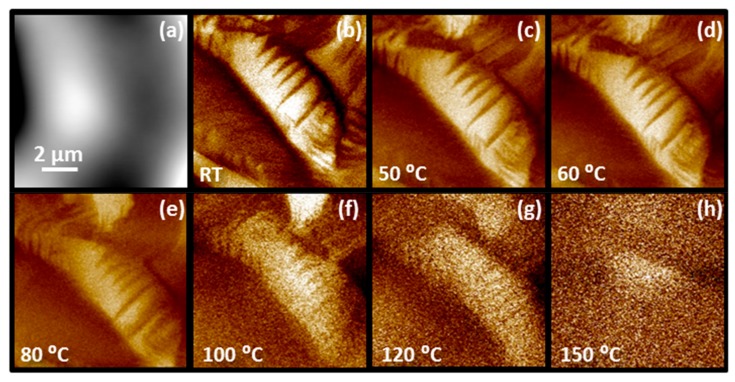
(**a**) Topography and out-of-plane PFM amplitude images of the same area (**b**) before and (**c**–**h**) after heating (drive AC signal: 5 V, 310 kHz).

As already mentioned, the domain structure of ceramics (this work) and single crystals [12] differ at ambient temperature. Therefore, the question of how the structure is upon heating appeared. The evolution of the initial domain structure with temperature is shown in Figure 5. The area for this experiment was chosen so that the micrometre-large wedge domains are clearly visible in the PFM amplitude image. Note that approximately 10 different areas were investigated at increasing temperature and a similar dependence was observed in all cases. By increasing the temperature, the domain structure is visible below 80 °C and it starts disappearing at ~100 °C, which coincides with the dielectric permittivity peak at ~98 °C, shown in Figure 1c. The temperature evolution of domain structure correlates well with the macroscopic dielectric permittivity versus temperature measurement, indicating that the observed domain structure resembles the domain structure of the whole sample and not just of the surface layer. At 150 °C, the domains cannot be discerned anymore. In contrast, between 60 and 80 °C [12] the wedge domains in the PFN single crystals have already disappeared.

**Figure 6:**
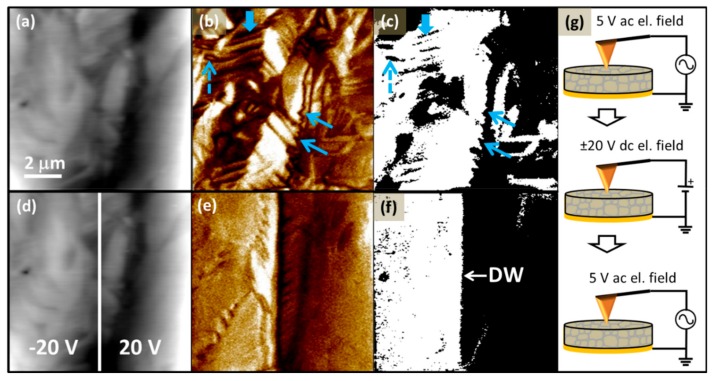
Topography (**a**,**d**), PFM out-of-plane amplitude (**b**,**e**), and phase (**c**,**f**) images (drive AC signal: 5 V, 350 kHz) before (up) and after (down) in situ poling with ±20 V at ambient temperature. (**g**) Schematic presentation of in situ poling experiment. Areas of different poling regimes are shown in (**d**). Examples of wedge, lamellar-like, and irregularly shaped domains are marked by solid, thick solid, and dashed arrows, respectively. Created DW is marked by an arrow in (**f**).

In this study, the switching behaviour of the ferroelectric domains in the PFN material was investigated for the first time. The PFM amplitude and phase hysteresis loops are shown in Figure 2c,d, indicating a good domain-switching ability. The domain-nucleation voltages ±*V*_DN_ are ~9 V. The domain-switching behaviour was further studied by in situ poling, as shown in Figure 6. First, the virgin area was scanned with an AC field of 5 V (Figure 6a–c). After scanning, the in situ poling was performed, meaning that the area was scanned with the PFM tip under a DC field with an amplitude of ±20 V, which is much higher than the ±*V*_DN_ of the sample. The “read” process of the locally switched area was performed by applying similar scanning conditions as prior to the application of a DC voltage. We were able to in situ pole the scanned area almost perfectly (as seen in Figure 6d–f), providing evidence that all types of domains, i.e., the wedge-like, lamellar-like, and irregularly shaped domains, can be easily switched. By poling half the area with 20 V and the other half with −20 V, two domains separated by a straight DW were created (Figure 6f), demonstrating that PFN is a promising material for domain-wall engineering.

## 4. Conclusions

In this study, ferroelectric domains with different morphologies and sizes—from a few hundred nanometres to micrometres—were observed by piezoresponse force microscopy. The ferroelectric domain structure is in agreement with its long-range ferroelectric behaviour, observed by macroscopic *P*–*E* measurements. The results are supported by scanning transmission electron microscopy, which revealed that the samples are chemically homogeneous. The characteristic domain structure can be well correlated between the PFM, SEM, and STEM techniques, confirming that these methods are complementary. By increasing the temperature, the ferroelectric domain structure is visible up to ~80 °C, and then it starts disappearing at ~100 °C, which coincides with the dielectric permittivity peak at 98 °C. Furthermore, custom-made domain walls can be created by poling neighbouring areas with voltages of opposite signs at ambient temperature, providing a proof-of-concept for domain-wall engineering in PFN ceramics.

## Supplementary Materials

The following are available online at https://www.mdpi.com/1996-1944/12/8/1327/s1, Figure S1: FE-SEM micrographs of (a) polished and (b) fractured surface, Figure S2: STEM image of grains in PFN sample, sintered at 1000 °C with the corresponding EDXS elemental maps showing chemically homogenous polycrystalline sample without secondary phases, Figure S3: XRD profiles of the (200), (220), and (222) pseudocubic reflections (corresponding only to Cu Kα1) for PFN obtained after Rietveld refinement of the room-temperature XRD data, considering the (a) Cm, (b) R3mR, and (c) P4mm space groups. The black dots represent the observed data, the red line is the calculated profile, and the grey bottom line is the difference between the calculated and experimental (observed) profiles. The marked 2θ positions show the allowed Bragg peaks, Figure S4: The observed (dots), calculated (red line), and difference (grey bottom line) profiles obtained after Rietveld refinement of PFN using the monoclinic Cm space group. The marked 2θ positions below the profile show the allowed Bragg peaks for Cu Kα1, Table S1: Refined structural parameters for PFN using different space groups: *Cm, R*3*mR*, and *P*4*mm.*
