# Supplementary material for: Structure and Dynamics of Ferroelectric Domains in Polycrystalline Pb(Fe1/2Nb1/2)O3
Source: Materials (Basel). 2019 Apr 23;12(8):1327. doi: 10.3390/ma12081327 (PMC6515442; doi:10.3390/ma12081327)
Supplement: Supplementary file 1 [file materials-12-01327-s001.pdf]

materials

Article

Structure and Dynamics of Ferroelectric Domains in Polycrystalline $Pb(Fe_{1/2}Nb_{1/2})O_3$

Hana Ursic [1,2,*], Andreja Bencan [1,2], Uros Prah [1,2], Mirela Dragomir [1,3] and Barbara Malic [1,2]

1 Electronic Ceramics Department, Jozef Stefan Institute, Jamova cesta 39, 1000 Ljubljana, Slovenia; andreja.bencan@ijs.si (A.B.); uros.prah@ijs.si (U.P.); mirela85.dragomir@gmail.com (M.D.); barbara.malic@ijs.si (B.M.)
2 Electronic Ceramics Department, Jozef Stefan International Postgraduate School, Jamova cesta 39, 1000 Ljubljana, Slovenia
3 Department of Chemistry and Chemical Biology, McMaster University, Hamilton, ON L8S 4K1, Canada
* Correspondence: hana.ursic@ijs.si

Supplementary Material

Supplement S1. Microstructural Characterisation of PFN Ceramic Samples

The FE-SEM images of the polished and fractured surface reveal dense, homogeneous, and uniform microstructure as shown in Figure S1. No secondary phases were observed, which was additionally confirmed also by STEM/EDXS analysis (Figure S2).

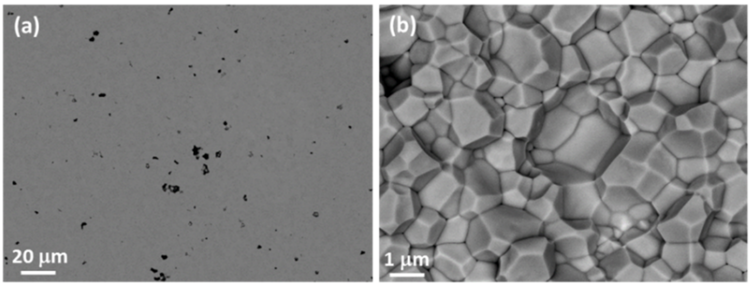

Figure S1. FE-SEM micrographs of (**a**) polished and (**b**) fractured surface.

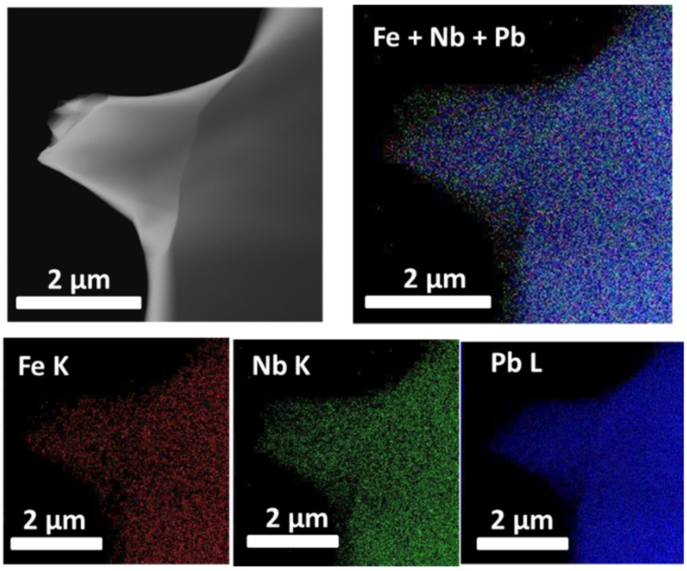

Figure S2. STEM image of grains in PFN sample, sintered at 1000 °C with the corresponding EDXS elemental maps showing chemically homogenous polycrystalline sample without secondary phases.

Supplement S2. Determination of the Phase Composition by Rietveld Refinement

At room temperature, PFN adopts the monoclinic phase with the space group *Cm* [1–5]. However, some reports also suggest a rhombohedral phase with the space group *R*3*m* [6, 7]. One study [8] even considered a tetragonal structure. To clarify which space group PFN prepared in this study adopts, the prepared PFN sample was analysed by Rietveld refinement of the room-temperature XRD data. Along with the monoclinic *Cm* (ICSD 88357), rhombohedral *R*3*mR* (ICSD 90490) and tetragonal *P*4*mm* (ICSD 88356) space groups were also considered for the fit.

Rietveld refinement analysis of the diffraction data of our samples was performed with Topas (version 6, Bruker, AXS, Karlsruhe, Germany) software. The fundamental parameters approach [9] was used to describe the peak profiles, while the background was estimated using a 5th order Chebychev polynomial. The sample displacement, lattice parameters, scale factor, background, strain, asymmetry [10], thermal displacement parameters, and atomic coordinates were stepwise refined to obtain a calculated diffraction profile that best-fit the experimental pattern. All the occupancies were fixed at nominal composition and kept constant during refinement. Finally, the quality of the fit was assessed from the fit parameters such as R_{wp}, R_p, R_{exp}, R_B, and *G.O.F.* [11]. The agreement factors of the Rietveld analysis along with the cell parameters are given in table S1, while the Rietveld fits plotted for the (200), (220), and (222) pseudocubic profiles are shown in Figure S3.

Table S1. Refined structural parameters for PFN using different space groups: *Cm*, *R*3*mR*, and *P*4*mm*.

Space Group	Unit Cell Parameters	R_{wp}	R_{exp}	R_p	G.O.F. (χ^2)	R_B
Cm	*a* (Å) = 5.67920(8)<br>*b* (Å) = 5.67239(9)<br>*c* (Å) = 4.01556(5)<br>β (°) = 89.8830(11)	6.012	5.528	4.535	1.087	1.373
*R*3*mR*	*a* (Å) = 4.013879(16)<br>α (°) = 89.9241(3)	6.933	5.531	5.197	1.253	3.152
*P*4*mm*	*a* (Å) = 4.01333(2)<br>*c* (Å) = 4.01552(7)	7.921	5.530	5.813	1.432	3.751

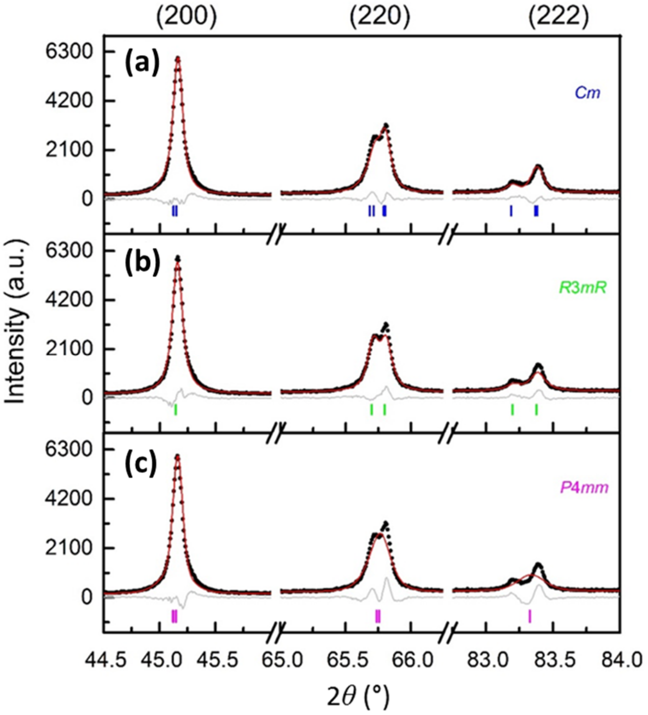

Figure S3. XRD profiles of the (200), (220), and (222) pseudocubic reflections (corresponding only to Cu Kα_1) for PFN obtained after Rietveld refinement of the room-temperature XRD data, considering

the (**a**) Cm, (**b**) R3mR, and (**c**) P4mm space groups. The black dots represent the observed data, the red line is the calculated profile, and the grey bottom line is the difference between the calculated and experimental (observed) profiles. The marked 2θ positions show the allowed Bragg peaks.

Looking at Figure S3, it is very easy to exclude the *P*4*mm* space group since the pseudocubic (222) reflection has to be a singlet for this space group, but in this case it is a doublet. The best agreement factors and matching between the observed and calculated profile were obtained with a monoclinic *Cm* phase with the unit-cell parameters: a = 5.67920(8) Å, b = 5.67239(9) Å, c = 4.01556(5) Å, and β = 89.8830(11)°. The Rietveld refined value for the thermal displacement parameter of Pb was anomalously large (3.03 Å^2), but comparable to the values reported by other researchers [3–5]. The reason is probably the presence of displacive disorder on the Pb-site. The whole observed (dots), calculated (red line), and difference (grey bottom line) profiles obtained after Rietveld refinement of PFN using the monoclinic *Cm* space group are shown in Figure S4. A very satisfactory Rietveld fit was also obtained when considering the *R*3*mR* space group (Table S1). As shown in Figure S3, it is hard to discern between the *Cm* and *R*3*mR* space groups based only on the Bragg reflections. The only notable difference between the two structures is the (200) reflection, which is a doublet for the *Cm* space group, but a singlet for the *R*3*mR* space group. No obvious splitting of this peak is observed here, but a careful examination of the full width at half maximum (FWHM) of this peak revealed a higher peak width than the (220) and the (222) peaks. This result is not supporting the assumption of (200) being a singlet since the FHWM of this peak does not follow the Caglioti relationship for the 2θ dependence of the peak width [3,12]. Therefore, the unusual broadening of the (200) peak can only be explained by a splitting of two reflections, which is consistent with the *Cm* space group and excludes the *R*3*mR* space group.

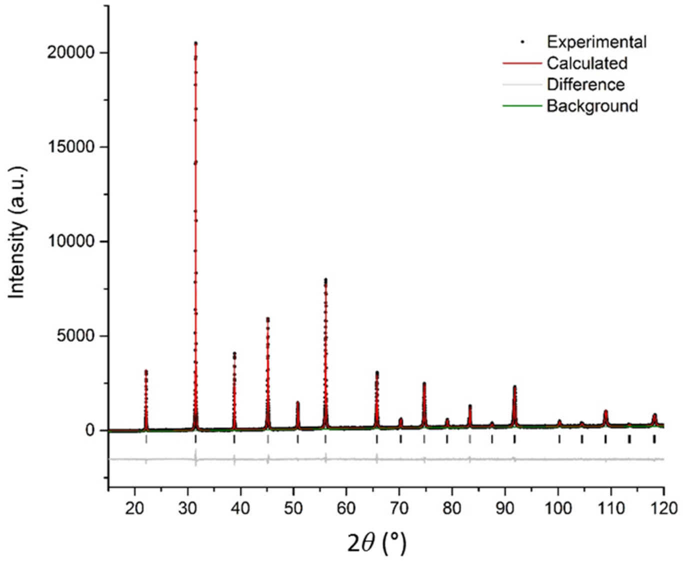

Figure S4. The observed (dots), calculated (red line), and difference (grey bottom line) profiles obtained after Rietveld refinement of PFN using the monoclinic Cm space group. The marked 2θ positions below the profile show the allowed Bragg peaks for Cu Kα_1.

References

1. Bonny, V.; Bonin, M.; Sciaub, P.; Schenk, K. J.; Chapuis, G.; Phase transitions in disordered lead iron niobate: X-ray and synchrotron radiation diffraction experiments. *Solid State Commun.* **1997**, *102*, 347–352.
2. Lampis, N.; Sciau, P.; Lehmann, A. G.; Rietveld refinements of the paraelectric and ferroelectric structures of $PbFe_{0.5}Nb_{0.5}O_3$. *J. Phys. Condens Matter.* **1999**, *11*, 3489–3500.
3. Singh, S. P.; Pandey, D.; Yoon, S.; Baik, S.; Shin, N.; Evidence for monoclinic crystal structure and negative thermal expansion below magnetic transition temperature in $Pb(Fe_{1/2}Nb_{1/2})O_3$. *Appl. Phys. Lett.*, **2007**, *90*, 242915 1–3.

4. Sim, H.; Peets, D. C.; Lee, S.; Lee, S.; Kamiyama, T.; Ikeda, K.; Otomo, T.; Cheong, S. W.; Park, J. G.; High-resolution structure studies and magnetoelectric coupling of relaxor multiferroic $Pb(Fe_{0.5}Nb_{0.5})O_3$. *Phys. Rev. B* **2014**, *90*, 1–8.
5. Matteppanavar, S.; Rayaprol, S.; Singh, K.; Reddy, V. R.; Angadi, B.; Evidence for magneto-electric and spin–lattice coupling in $PbFe_{0.5}Nb_{0.5}O_3$ through structural and magneto-electric studies. *J. Mater. Sci.* **2015**, *50*, 4980–4993.
6. Ivanov, S. A.; Tellgren, R.; Rundlof, H.; Thomas, N. W.; Ananta, S.; Investigation of the structure of the relaxor ferroelectric $Pb(Fe_{1/2}Nb_{1/2})O_3$ by neutron powder diffraction. *J. Phys. Condens. Matter.* **2000**, *12*, 2393–2400.
7. Mabud, S. A.; X-ray and neutron diffraction studies of lead iron niobate ceramics and single crystals. *Phase Trans.*, **1984**, *4*, 183–200.
8. Darlington, C. N. W.; Studies of transitions in ordered and disordered perovskites: x-ray and Mössbauer scattering experiments. *J. Phys.: Condens. Matter.* **1991**, *3*, 4173–4185.
9. Cheary, R. W.; Coelho, A. A.; Fundamental parameters approach to X-ray line-profile fitting. *J. Appl. Crystallogr.* **1992**, *25*, 109–1021.
10. Stephens, P. W.; Phenomenological model of anisotropic peak broadening in powder diffraction. *J. Appl. Cryst.* **1999**, *32*, 281–289.
11. David, W. I. F.; Powder diffraction: least-squares and beyond, *J. Res. Natl. Inst. Stand. Technol.* **2004**, *109*, 107–123.
12. Caglioti, G.; Paoletti, A.; Ricci, F. P.; Choice of collimators for a crystal spectrometer for neutron diffraction, *Nucl. Instrum.* **1958**, *3*, 223–238.

© 2019 by the authors. Submitted for possible open access publication under the terms and conditions of the Creative Commons Attribution (CC BY) license (http://creativecommons.org/licenses/by/4.0/).
